# In vitro and in vivo pharmacological characterization of a neuropeptide S tetrabranched derivative

**DOI:** 10.1002/prp2.108

**Published:** 2015-01-05

**Authors:** Chiara Ruzza, Anna Rizzi, Davide Malfacini, Alice Pulga, Salvatore Pacifico, Severo Salvadori, Claudio Trapella, Rainer K Reinscheid, Girolamo Calo, Remo Guerrini

**Affiliations:** 1 Department of Medical Sciences, Section of Pharmacology and National Institute of Neuroscience, University of Ferrara44121, Ferrara, Italy; 2 Department of Chemical and Pharmaceutical Sciences and LTTA, University of Ferrara44121, Ferrara, Italy; 3 Department of Pharmaceutical Sciences, University of California IrvineIrvine, California, 92697

**Keywords:** Calcium mobilization, locomotor activity, mice, neuropeptide S, neuropeptide S receptor, PWT-NPS, righting reflex

## Abstract

The peptide welding technology (PWT) is a novel chemical strategy that allows the synthesis of multibranched peptides with high yield, purity, and reproducibility. With this approach, a tetrabranched derivative of neuropeptide S (NPS) has been synthesized and pharmacologically characterized. The in vitro activity of PWT1-NPS has been studied in a calcium mobilization assay. In vivo, PWT1-NPS has been investigated in the locomotor activity (LA) and recovery of the righting reflex (RR) tests. In calcium mobilization studies, PWT1-NPS behaved as full agonist at the mouse NPS receptor (NPSR) being threefold more potent than NPS. The selective NPSR antagonists [^*t*^Bu-D-Gly^5^]NPS and SHA 68 displayed similar potency values against NPS and PWT1-NPS. In vivo, both NPS (1–100 pmol, i.c.v.) and PWT1-NPS (0.1–100 pmol, i.c.v.) stimulated mouse LA, with PWT1-NPS showing higher potency than NPS. In the RR assay, NPS (100 pmol, i.c.v.) was able to reduce the percentage of mice losing the RR after diazepam administration and their sleep time 5 min after the i.c.v. injection, but it was totally inactive 2 h after the injection. On the contrary, PWT1-NPS (30 pmol, i.c.v.), injected 2 h before diazepam, displayed wake-promoting effects. This PWT1-NPS stimulant effect was no longer evident in mice lacking the NPSR receptor. The PWT1 technology can be successfully applied to the NPS sequence. PWT1-NPS displayed in vitro a pharmacological profile similar to NPS. In vivo PWT1-NPS mimicked NPS effects showing higher potency and long-lasting action.

## Introduction

Neuropeptide S (NPS, human sequence SFRNGVGTGMKKTSFQRAKS) was identified as the endogenous ligand of a previously orphan G proteins coupled receptor (GPCR) now named NPS receptor (NPSR; Xu et al. 2004). In cells expressing the recombinant NPSR, NPS displayed high affinity and stimulated calcium mobilization and cAMP accumulation suggesting Gq and Gs coupling (Reinscheid et al. 2005). In vivo, NPS has been shown to control several biological functions in rodents including stress and anxiety, locomotor activity (LA), wakefulness, food intake and gastrointestinal functions, memory processes, and drug abuse (for a review see Guerrini et al. (2010)).

The peptide welding technology (PWT) is a recently developed chemical strategy that allows the synthesis of multibranched peptides with extraordinary high yield, purity, and reproducibility (Guerrini et al. 2014). The PWT has been successfully applied to nociceptin/orphanin FQ (N/OFQ) (Rizzi et al. 2014) and tachykinin peptides (Ruzza et al. 2014). It has been demonstrated that these PWT compounds maintain in vitro the same pharmacological profile of the native peptides but display in vivo higher potency associated with longer lasting action.

In the present work, the PWT derivative of NPS has been synthesized and pharmacologically characterized in vitro, in the calcium mobilization assay performed in human embryonic kidney (HEK 293) cells stably transfected with the murine NPSR (HEK293_mNPSR_), and in vivo in mice in the LA and in the righting reflex (RR) tests. In the latter test, the selectivity of action of PWT1-NPS has been assessed by using NPSR knockout (NPSR(−/−)) mice.

## Materials and Methods

### Synthesis of PWT1-NPS

**Figure 1:**
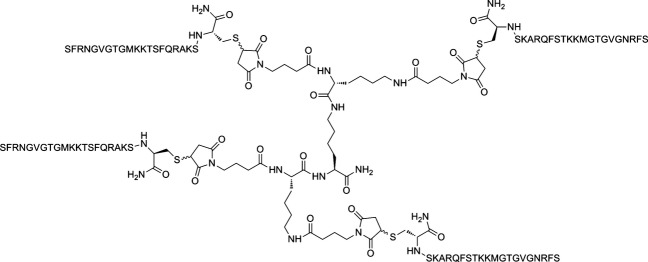
Chemical formula of PWT1-NPS.

PWT derivative of NPS was prepared by using a convergent synthetic approach and methodology previously applied for the synthesis of PWT derivatives of N/OFQ peptide (Guerrini et al. 2014). Firstly, [Cys^21^]NPS was synthesised by solid phase method with an automatic solid phase peptide synthesizer Syro II (Biotage, Uppsala, Sweden) using Fmoc/tBu chemistry (Benoiton 2005). The resin 4-(2′,4′-dimethoxyphenyl-Fmoc-aminomethyl)-phenoxyacetamido-norleucyl-MBHA (Rink amide MBHA resin) was used as a solid support. The resin was treated with 40% piperine/*N*,*N*-dimethylformamide (DMF) and linked with Fmoc-Cys(Trt)-OH by using [*O*-(7-azabenzotriazol-1-yl)-1,1,3,3-tetramethyluronium hexafluorophosphate] (HATU) as the coupling reagent. The following Fmoc amino acids were sequentially coupled to the growing peptide chain: Fmoc-Ser(tBu)-OH, Fmoc-Lys(Boc)-OH, Fmoc-Ala-OH, Fmoc-Arg(Pmc)-OH, Fmoc-Gln(Trt)-OH, Fmoc-Phe-OH, Fmoc-Ser(tBu)-OH, Fmoc-Thr(tBu)-OH, Fmoc-Lys(Boc)-OH, Fmoc-Lys(Boc)-OH, Fmoc-Met-OH, Fmoc-Gly-OH, Fmoc-Thr(tBu)-OH, Fmoc-Gly-OH, Fmoc-Val-OH, Fmoc-Gly-OH, Fmoc-Asn(Trt)-OH, Fmoc-Arg(Pmc)-OH, Fmoc-Phe-OH, Fmoc-Ser(tBu)-OH. All the Fmoc amino acids (4 equiv) were coupled to the growing peptide chain by using HATU (4 equiv) in DMF in the presence of an equimolar concentration of 4-methylmorpholine (NMM), and the coupling reaction time was 1 h. To improve the analytical profile of the crude peptide, capping with acetic anhydride (0.5 mol/L/DMF) in the presence of NMM (0.25mol/L/DMF) (3:1 v/v; 2 mL/0.2 g of resin) was performed at any step. About 40% of Piperidine/DMF was used to remove the Fmoc. The protected peptide-resin was treated with reagent B (Sole and Barany 1992) (trifluoroacetic acid (TFA)/H_2_O/phenol/triisopropylsilane 88: 5: 5: 2; v/v; 10 mL/0.2 g of resin) for 1.5 h at room temperature. After filtration of the resin, the solvent was concentrated in vacuum and the residue triturated with ether. Crude [Cys^21^]NPS was purified by preparative reversed-phase high-performance liquid chromatography (HPLC) using a Water Delta Prep 3000 (Meadow Instrumentation, Bristol, WI, USA) system with a Jupiter column C_18_ (250 × 30 mm, 300 A, 15 *μ*m spherical particle size). The column was perfused at a flow rate of 20 mL/min with a mobile phase containing solvent A (5%, v/v, acetonitrile in 0.1% TFA), and a linear gradient from 0 to 60% of solvent B (60%, v/v, acetonitrile in 0.1% TFA) over 25 min for the elution of peptides. Purified [Cys^21^]NPS was reacted in solution with PWT2 core in a classical thio-Michael reaction using experimental conditions previously optimized for the synthesis of N/OFQ tetrabranched derivatives (Guerrini et al. 2014). Analytical HPLC analyses were performed on a Beckman 116 liquid chromatography equipped with a Beckman 166 diode array detector. Analytical purity of [Cys^21^]NPS and PWT2-NPS was determined using a Luna C_18_ column (4.6 × 100 mm, 3 *μ*m particle size) with the above solvent system (solvents A and B) programed at a flow rate of 0.5 mL/min using a linear gradient from 0% to 600% B over 25 min. Final product showed ≥95% purity when monitored at 220 nm. Molecular weight of PWT1-NPS was in accord with the expected molecular formula. PWT1-NPS chemical structure is shown in Fig. 1.

### Calcium mobilization assay

HEK293_mNPSR_ cells were maintained in Dulbecco’s Medium (DMEM) supplemented with 10% fetal bovine serum, 2 mmol/L l-glutamine, and 100 mg/L hygromycin. The cells were cultured at 37°C in 5% CO_2_ humidified air and seeded at a density of 50,000 cells/well into poly-d-Lysine coated 96-well black, clear-bottom plates. The following day, the cells were incubated with a medium supplemented with 2.5 mmol/L probenecid, 3 *μ*mol/L calcium-sensitive fluorescent dye Fluo-4 am, and 0.01% pluronic acid, for 30 min at 37°C. After that time the loading solution was aspirated and 100 *μ*L/well of assay buffer Hank’s Balanced Salt Solution (HBSS) supplemented with 20 mmol/L 4-(2-hydroxyethyl)-1-piperazineethanesulfonic acid (HEPES), 2.5 mmol/L probenecid, and 500 *μ*mol/L Brilliant Black (Sigma-Aldrich, St. Louis, MO) was added. Concentrated solutions (1 mmol/L) of NPS, [Cys^21^]NPS-NH_2_, PWT1-NPS, and [^*t*^Bu-D-Gly^5^]NPS were made in bidistilled water. SHA 68 was solubilized in dimethyl sulfoxide (DMSO) (10 mmol/L). Serial dilutions were carried out in HBSS/HEPES (20 mmol/L) buffer (containing 0.02% bovine serum albumin (BSA) fraction V). After placing both plates (cell culture and compound plate) into the FlexStation II, fluorescence changes were measured at 37°C. Online additions were carried out in a volume of 50 *μ*L/well. Using previously validated protocols (Guerrini et al. 2009b; Ruzza et al. 2010), in antagonist-type experiments the compounds under study were preincubated for 24 min before agonist addition. To facilitate drug diffusion into the wells in antagonist type experiments, three cycles of mixing (25 *μ*L from each well moved up and down three times) were performed immediately after antagonist injection to the wells. Maximum change in fluorescence, expressed in percent of baseline fluorescence, was used to determine agonist response.

### In vivo studies

All experimental procedures adopted for in vivo studies were as humane as possible and complied with the European Communities Council directives (2010/63/EU) and national regulations (D.L. 116/92). The present study was approved by the Ethical Committee for the Use of Laboratory Animals (CEASA) of the University of Ferrara and by the Italian Ministry of Health (authorization number 75-76/2013-B). This research was reported following the ARRIVE guidelines (Kilkenny et al. 2010) and complies with the prevailing standards of animal welfare embodied in UK laws governing animal experimentation. Male CD-1 mice (weight 28–35 g; Harlan, Udine, Italy) and male NPSR(−/−) and NPSR(-/-) mice congenic to CD-1 strain (8 weeks old, bred in animal facility of the Section of Pharmacology of the University of Ferrara) were used in a total number of 146. They were housed in 267 × 207 × 140 mm cages (Tecniplast, Buguggiate, Italy), five mice/cage, under standard conditions (22°C, 55% humidity, 12 h light–dark cycle, lights on 7.00 am) with food (standard diet; Mucedola, Settimo Milanese, Italy), and water ad libitum for at least 15 days before experiments began. Each cage was provided with a mouse red house (Tecniplast, Buguggiate, Italy) and nesting materials. Each animal was used only once. NPS and PWT1-NPS were injected intracerebroventricularly (i.c.v.). I.c.v. injections (2 *μ*L per mouse) were given under light isoflurane anesthesia, into the left ventricle according to the procedure described by Laursen and Belknap (1986) and routinely adopted in our laboratory (Rizzi et al. 2008).

### LA assay

Experiments were performed during the light cycle (between 09.00 and 13.00) according to previously reported procedures (Guerrini et al. 2009b). For these experiments, the ANY-maze video tracking system was used (Ugo Basile, Varese, Italy; application version 4.52c Beta). Mice were positioned in a square plastic cage (40 cm × 40 cm), one mouse per cage. Four mice were monitored in parallel. Mouse’s horizontal activity was monitored by a camera, while vertical activity was measured by an infrared beam array. Animals’ locomotion was recorded for 120 min. The parameters measured were cumulative distance travelled (total distance in m that the animal travelled during the test), immobility time (the animal is considered immobile when 90% of it remains in the same place for a minimum of 2.5 sec), and the number of rearings (the number of beam breaks due to vertical movements; this input is triggered when the beam is interrupted for a minimum of 200 msec). NPS (1–100 pmol) and PWT1-NPS (0.1–100 pmol) were injected i.c.v. 15 min before starting the test.

### RR assay

This assay was performed according to the procedures previously described in detail (Rizzi et al. 2008). Briefly, mice were given an i.p. injection of diazepam (15 mg/kg; Sigma-Aldrich). When the animals lost the RR, they were placed in a plastic cage and the time was recorded by an expert observer blind to drug treatments. Animals were judged to have regained the RR response when they could right themselves three times within 30 sec. Sleep time is defined as the amount of time between the loss and regaining of the RR and was rounded to the nearest minute. NPS (100 pmol) and PWT1-NPS (30 pmol) were injected i.c.v. 5 min, 2, and 4 h before diazepam administration. These time points were selected because previous studies demonstrated NPS effectiveness at 5 min (e.g., Rizzi et al. 2008) and unpublished results indicated lack of effect of NPS after 2 h from injection. In the experiments performed with NPSR(-/-) and NPSR(−/−) mice, PWT1-NPS was injected 2 h before diazepam.

### Drugs and reagents

NPS was synthesized according to published methods (Guerrini et al. 2009b) using Fmoc/tBu chemistry with a SYRO XP multiple peptide synthesizer (MultiSyntech, Witten, Germany). Crude peptide was purified by preparative reversed-phase HPLC and the purity checked by analytical HPLC and mass spectrometry using an ESI Micromass ZMD-2000 mass spectrometer (Waters Corporation, Milford, MA). SHA 68 was synthesized using the procedures described by Okamura et al. (2008). For the in vitro studies NPS, PWT1-NPS, and [^*t*^Bu-D-Gly^5^]NPS were dissolved in water, while SHA 68 was dissolved in DMSO. For the in vivo experiments, the vehicle used for injecting NPS and PWT1-NPS was saline.

### Data analysis and terminology

The pharmacological terminology adopted in this paper is consistent with IUPHAR recommendations (Neubig et al. 2003). In vitro data were expressed as mean ± SEM of at least four independent experiments made in duplicate. Maximum change in fluorescence, expressed as percent over the baseline fluorescence, was used to determine agonist response. Nonlinear regression analysis using GraphPad Prism software (v.5.0; GraphPad, La Jolla, CA) allowed logistic interactive fitting of the resultant responses and the calculation of agonist potencies and maximal effects. Agonist potencies were given as pEC_50_ (the negative logarithm to base 10 of the molar concentration of an agonist that produces 50% of the maximal possible effect). [^*t*^Bu-D-Gly^5^]NPS and SHA 68 antagonist properties were evaluated in inhibition response curve experiments versus a fixed concentration of NPS and PWT1-NPS approximately corresponding to its EC_80_; the antagonist potency was expressed as pK_B_ derived from the following equation:

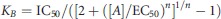
where IC_50_ is the concentration of antagonist that produces 50% inhibition of the agonist response, [*A*] is the concentration of agonist, EC_50_ is the concentration of agonist producing a 50% maximal response, and n is the Hill coefficient of the concentration response curve to the agonist (Kenakin 2004). In vivo data are expressed as mean ± sem of n animals. Data were analyzed using one-way analysis of variance (ANOVA) followed by the Dunnett’s post hoc test or two-way ANOVA followed by the Bonferroni’s post hoc test, as specified in figure legends. Differences were considered statistically significant when *P* < 0.05.

## Results

### Calcium mobilization assay

**Figure 2:**
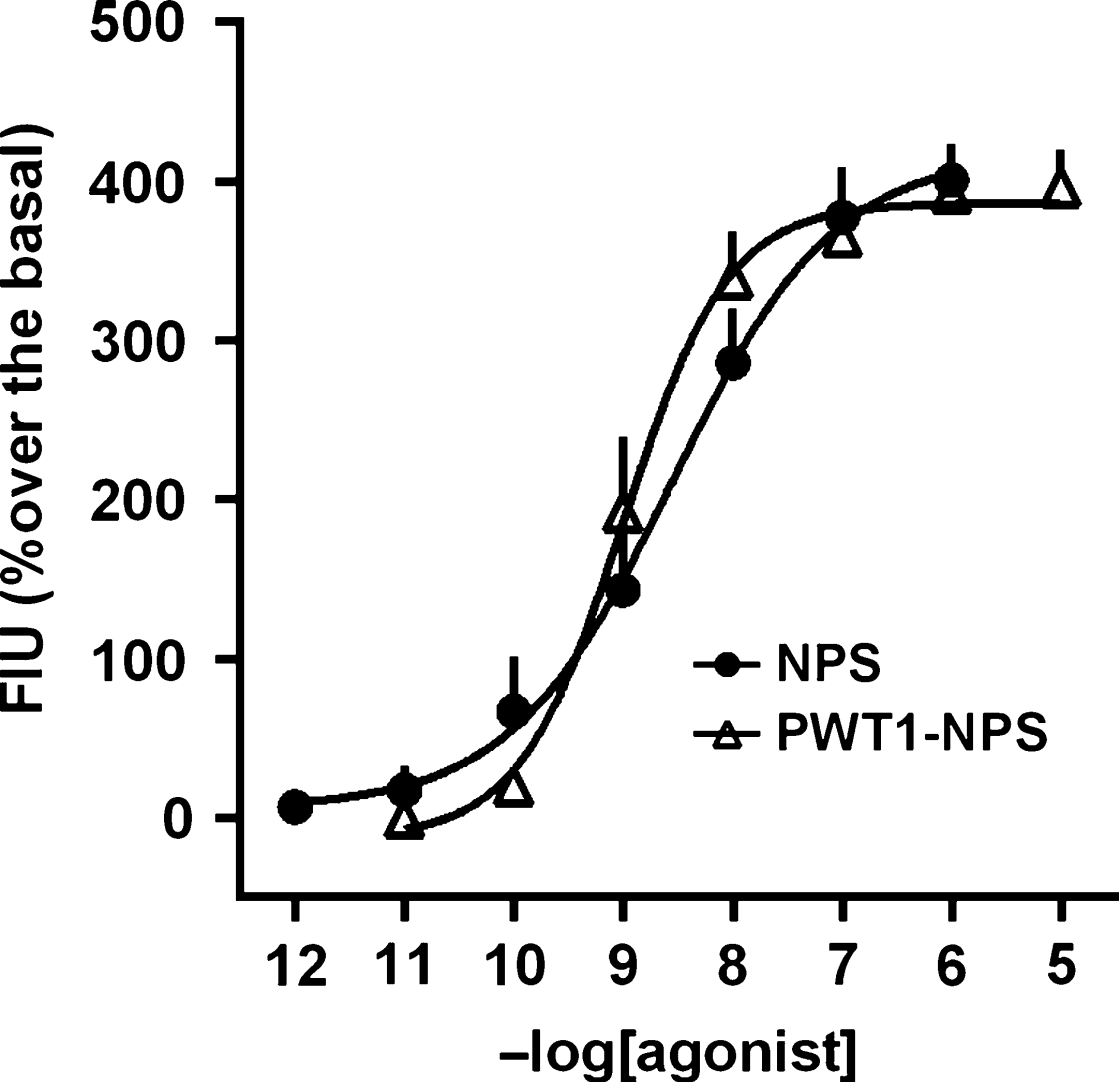
Calcium mobilization assay performed in HEK293 cells expressing the mouse NPSR receptor. Concentration response curve to NPS and PWT1-NPS Data are mean ± SEM of three experiments made in duplicate.

**Figure 3:**
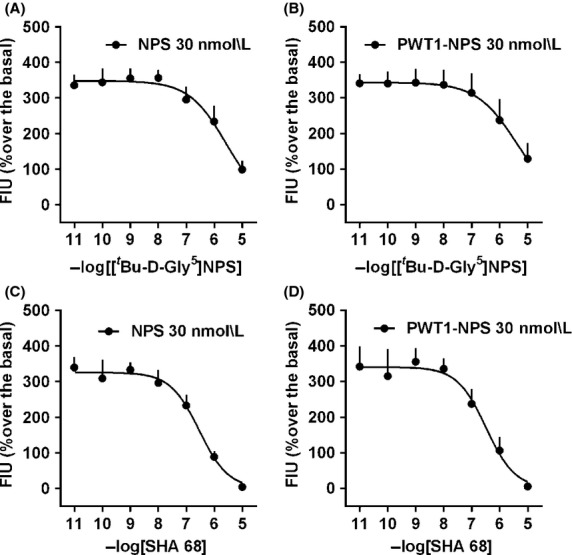
Calcium mobilization assay performed in HEK293 cells expressing the mouse NPSR receptor. Inhibition response curve to [^*t*^Bu-D-Gly^5^]NPS (A and B) and SHA 68 (C and D) against the stimulatory effect of NPS (A and C) and PWT1-NPS (B and D). Data are mean ± SEM of three experiments made in duplicate.

In HEK293_mNPSR_ cells, NPS increased intracellular calcium levels in a concentration-dependent manner with pEC_50_ and *E*_max_ values of 8.55 (8.09–9.00) and 417 ± 34% over the basal levels, respectively. As shown in Figure2, PWT1-NPS stimulated calcium mobilization producing similar maximal effects as the natural peptide but being threefold more potent (pEC_50_ = 8.99 [8.68–9.31]). The stimulatory effects produced by NPS and PWT1-NPS were challenged with the selective NPSR antagonists [^*t*^Bu-D-Gly^5^]NPS and SHA 68 in inhibition response experiments. [^*t*^Bu-D-Gly^5^]NPS inhibited the stimulatory effects evoked by NPS (30 nM) and PWT1-NPS (30 nM) with similar potency values (pK_B_ 6.89 [6.57–7.21] vs. NPS and 6.88 [6.41–7.35] vs. PWT1-NPS, Figure3A and B). Similarly, the nonpeptide NPSR antagonist SHA 68 blocked in a concentration-dependent manner the stimulant action of NPS and PWT1-NPS with similar potency (pK_B_ = 7.84 [6.97–8.71] and 7.99 [7.76–8.22], respectively, Fig.3C and D).

### LA test

**Figure 4:**
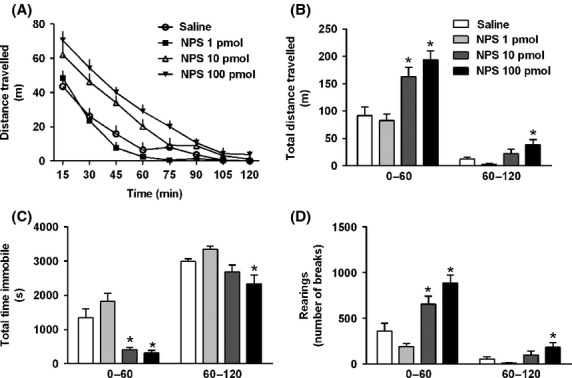
Mouse locomotor activity test. Dose–response curve to NPS (1–100 pmol, i.c.v., 15 min before starting the test). Time course of the distance travelled is shown in (A). According to one-way ANOVA followed by the Dunnett’s post hoc test, NPS elicited a statistically significant effect on the cumulative distance travelled (*F*_(3,27)_ = 17.79 0–60 min; *F*_(3,27)_ = 5.42 60–120 min, B), total time immobile (*F*_(3,27)_ = 12.57 0–60 min; *F*_(3,27)_ = 6.03 60–120 min, C), and number of rearings (*F*_(3,27)_ = 16.59 0–60 min; *F*_(3,27)_ = 3.96 60–120 min, D). Data are mean ± SEM of eight mice per group, *P < 0.05 vs. saline.

**Figure 5:**
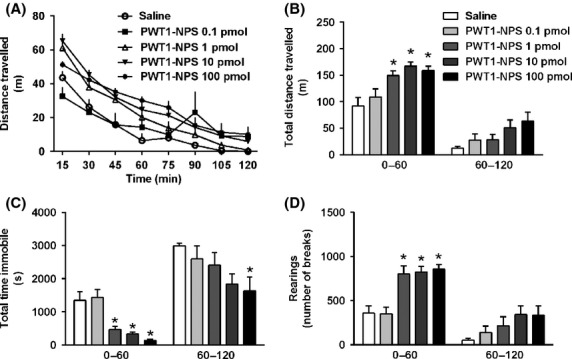
Mouse locomotor activity test. Dose–response curve to PWT1-NPS (0.1–100 pmol, i.c.v., 15 min before starting the test). Time course of the distance travelled is shown in panel A. According to one-way ANOVA followed by the Dunnett’s post hoc test, PWT1-NPS elicited a statistically significant effect on the cumulative distance travelled (*F*_(4,32)_ = 7.95 0–60 min, B), total time immobile (*F*_(4,32)_ = 13.49 0–60 min; *F*_(4,32)_ = 2.70 60–120 min, C), and number of rearings (*F*_(4,32)_ = 12.20 0–60 min, D). Data are mean ± SEM of 7–8 mice per group, *P < 0.05 vs. saline.

In the LA test, mouse horizontal and vertical activity were measured for 2 h after i.c.v. injection. Saline-treated mice explore the LA arena especially during the first hour of the experiment, while their LA resulted widely reduced during the second hour of test. As shown in Figure4, NPS (1–100 pmol) evoked, in a dose-dependent manner, a robust stimulatory effect on mouse LA by increasing the cumulative distance travelled by the animals and their number of rearings, and reducing the total immobility time. NPS was active from the 10 pmol dose and this effect lasted approximately for 1 h after the injection. During the second hour of the test, only the higher NPS dose (100 pmol) was able to produce statistically significant effects. PWT1-NPS (0.1–100 pmol) increased the distance travelled by the animals and their number of rearings, and reduced the total immobility time from the dose of 1 pmol, resulting in this test 10-fold more potent than NPS. Similar to NPS, the stimulatory effects evoked by PWT1-NPS were no longer evident during the second hour of the test. Only the highest dose of PWT1-NPS used (100 pmol) was able to reduce, in a statistically significant manner, the immobility time of mice during the second hour (Fig.5).

### Recovery of RR

**Figure 6:**
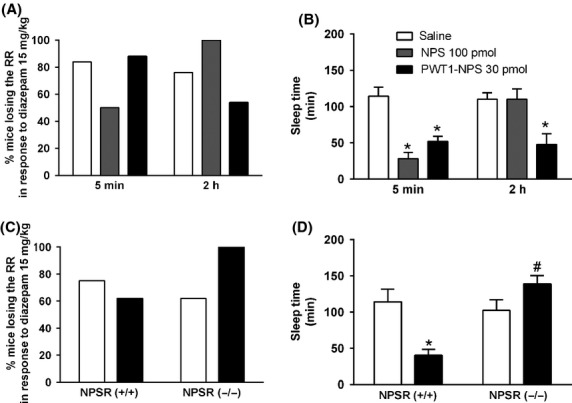
Recovery of righting reflex test performed in CD-1 mice (A and B) and in NPSR(-/-) and NPSR(−/−) mice (C and D). The percent of animals losing the RR after diazepam (15 mg/kg, i.p.) administration is shown in A and C, while B and D display mice sleep time. In CD-1, mice sleep time, two-way ANOVA followed by the Bonferroni’s post hoc test, revealed aneffect of NPS (100 pmol, i.c.v) and PWT1-NPS (30 pmol, i.c.v., *F*_(2,44)_ = 13.89), an effect of time (*F*_(2,44)_ = 5.60) and a significant interaction treatment × time (*F*_(2,44)_ = 7.25; B). Data are mean ± SEM of 8–9 mice per group, *P < 0.05 vs. saline. In NPSR(-/-) and NPSR(−/−) mice, two-way ANOVA followed by the Bonferroni’s post hoc test, revealed an effect of PWT1-NPS on mice sleep time (*F*_(1,21)_ = 9.47) and a significant interaction PWT1-NPS × genotype (*F*_(1,21)_ = 15.31, D). Data are mean ± SEM of 8–9 mice per group, *P < 0.05 vs. saline, #*P* < 0.05 vs. NPSR(-/-).

As demonstrated in previous studies, in the RR, 0.1 nmol NPS, injected i.c.v. 5 min before diazepam, was able to reduce the percentage of mice losing the RR and to reduce the sleep time of those mice responding to diazepam. This effect of NPS was no longer evident when NPS was injected 2 h before diazepam administration. On the contrary, PWT1-NPS (0.03 pmol, i.c.v.), given 5 min before diazepam, did not change the percentage of mice losing the RR but was able to significantly reduce the sleep time induced by diazepam. When PWT1-NPS was injected 2 h before diazepam administration it reduced both the percentage of mice losing the RR and the sleep time of those mice responding to diazepam (Fig.6A and B). PWT1-NPS was not able to produce any statistically significant effects 4 h after the injection (data not shown). Under the present experimental conditions, no differences were measured between NPSR(-/-) and NPSR(−/−) mice in terms of sensitivity to diazepam. PWT1-NPS (0.03 pmol, i.c.v., 2 h of pretreatment) elicited a robust wake-promoting effect in NPSR(-/-) mice; this action of PWT1-NPS was no longer evident in mice lacking the NPSR receptor (Fig.6C and D).

## Discussion and Conclusions

In the present study, the pharmacological profile of a tetrabranched derivative of NPS generated with the PWT has been investigated in vitro and in vivo.

The PWT is an innovative chemical strategy that allows the efficient synthesis of multibranched peptides. The chemical details of the PWT have been recently described (Guerrini et al. 2014) and PWT derivatives of N/OFQ (Rizzi et al. 2014) and tachykinins (Ruzza et al. 2014) have been generated and pharmacologically characterized. This technology is based on the extremely chemoselective reaction between a maleimide functionalized central core and a Cys residue that must be introduced into the peptide sequence. This attachment point is crucial for maintaining biological activity. Since previous structure activity studies indicate that the N-terminal region of NPS plays a crucial role for bioactivity (Bernier et al. 2006; Roth et al. 2006), the Cys residue has been added at the C terminus of NPS and [Cys^21^]NPS has been used for generating PWT1-NPS.

In the calcium mobilization assay, NPS produced a concentration-dependent increase in intracellular calcium levels, similar to previously reported data (Camarda et al. 2008a,b, 2009; Guerrini et al. 2009a,b). PWT1-NPS mimicked the stimulatory effect of NPS displaying similar maximal effects. Thus, PWT1-NPS behaved as a full NPSR agonist. Comparable results have been obtained with PWT derivatives of N/OFQ (Rizzi et al. 2014) and tachykinins (Ruzza et al. 2014). Thus, the PWT has no impact on the ability of the peptide sequence to bind and fully activate the receptor. As far as potency is concerned, PWT1-NPS resulted in the calcium mobilization assay threefold more potent than NPS. Interestingly, this is the first PWT derivative, so far evaluated, that displayed in this assay higher potency than the natural sequence. In fact, in this test, N/OFQ, SP, and NK-A were more potent than their PWT derivatives, while NK-B and PWT2-NKB displayed similar potency values (Rizzi et al. 2014; Ruzza et al. 2014). As discussed in detail in Rizzi et al. (2014) and in Ruzza et al. (2014), this loss of potency of PWT derivatives in the calcium mobilization assay has been ascribed to the fact that PWT derivatives of N/OFQ, SP, and NKA need longer time to occupy and fully activate their receptors than the natural peptide sequences. This slow interacting kinetic is not compatible to the rapid and transient nature of the calcium spike. As a consequence, the calcium assay tends to underestimate the potency of slow interacting ligands (Charlton and Vauquelin 2010). Based on these considerations, it can be speculated that the kinetic of interaction with NPSR is similar for NPS and PWT1-NPS and is probably faster than that of the other PWT derivatives so far characterized. Of note, both the peptidic NPSR antagonist [^*t*^Bu-D-Gly^5^]NPS (Guerrini et al. 2009a) and the nonpeptidic NPSR antagonist SHA 68 (Okamura et al. 2008) inhibited the stimulant effects of NPS and PWT1-NPS with the same potency, demonstrating that the mechanisms by which PWT1-NPS elicited the increase in intracellular calcium levels is the activation of NPSR.

In vivo, PWT1-NPS has been tested in the LA and in the RR. Several studies demonstrated that NPS increases mouse LA (Xu et al. 2004; Rizzi et al. 2008; Castro et al. 2009; Guerrini et al. 2009b; Paneda et al. 2009) and produces wake-promoting effects in the RR test (Rizzi et al. 2008; Camarda et al. 2009; Kushikata et al. 2011). Both knockout (Camarda et al. 2009; Duangdao et al. 2009; Zhu et al. 2010; Fendt et al. 2011; Ruzza et al. 2012a) and antagonism (Okamura et al. 2008; Camarda et al. 2009; Ruzza et al. 2010, 2012b; Kushikata et al. 2011) studies demonstrated that the stimulant and wake-promoting actions of NPS are due to the selective activation of NPSR. In line with previous findings, in the present study NPS increased mouse LA. PWT1-NPS mimicked NPS stimulant action showing 10-fold higher potency. In fact the lower dose of NPS producing statistically significant effects was 100 pmol while that of PWT1-NPS was 10 pmol. In the RR test, NPS 100 pmol evoked wake-promoting effects. PWT1-NPS, at the dose of 30 pmol, produced in the RR similar effects as those elicited by NPS. Thus, in mice, PWT1-NPS was able to elicit the same effects of NPS but being 3–10 more potent.

As far as the duration of action is concerned, no differences were measured in the LA assay between NPS and PWT1-NPS. Of note, mouse LA strongly decreases during the time course of the experiment. This is probably due to the mouse habituation to the open field. This phenomenon might be a bias for the interpretation of these results. In fact, under these experimental conditions, it is not clear if the lack of effects of NPS and PWT1-NPS during the second hour of the assay is imputable to the inactivation of the peptides or to a progressive reduction in animal sensitivity to their stimulant action. Considering this, LA is probably not the best assay to investigate the effects of long-lasting stimulant drugs. Interestingly, when tested in the RR test, PWT1-NPS displayed longer duration of action compared to NPS. In fact, at equieffective doses NPS and PWT1-NPS elicited similar and robust wake-promoting effects after 5 min from injection. However, PWT1-NPS, injected 2 h before diazepam, was still able to evoke statistically significant wake-promoting effects, while NPS resulted totally inactive at this time point. Thus, PWT1-NPS emerged in vivo not only more potent than NPS but also longer lasting. Similar findings were described for PWT derivatives of both N/OFQ (Rizzi et al. 2014) and SP (Ruzza et al. 2014). The available evidence suggests that these features may derive from an increased stability to enzymatic degradation and/or longer lasting binding to the receptor. In fact, an increased stability to enzymatic degradation has been demonstrated for multibranched derivatives of N/OFQ, enkephalins, and neurotensin (Bracci et al. 2003). No data are so far available regarding NPS susceptibility to enzymatic degradation. Therefore, future studies investigating the metabolic stability of NPS and PWT-NPS will be of high interest and utility. It is also possible that longer lasting binding to NPSR may contribute to increase the in vivo duration of action of PWT1-NPS. In fact, multivalent ligands, via multiple molecular mechanisms (Gestwicki et al. 2002), may display long-lasting target binding and this feature might be crucial for prolonging in vivo drug action (Vauquelin and Charlton 2010). In this regard, it is worthy of note that PWT derivatives of N/OFQ (Rizzi et al. 2014) and of SP (Ruzza et al. 2014) are less sensitive to wash in organ bath experiments compared to the natural peptide sequences. Unfortunately, similar kind of experiments could not be performed with PWT1-NPS since NPS-sensitive preparations useful for bioassay experiments are not described yet. However, it should be underlined that these considerations are rather speculative and well grounded hypotheses can be proposed only after direct investigation of NPS and PWT1-NPS metabolic stability and receptor-binding features. Available evidence suggests that increased in vivo potency associated with longer duration of action is a common feature of PWT derivatives. However, it is worth of note that quantitatively the amount of differences in potency and duration of action between the native peptide and its PWT derivative strongly depend on peptide sequence. In particular, PWT derivatives of N/OFQ resulted up to 40-fold more potent than N/OFQ and displayed extremely longer duration of action that is, from ∼20 min for N/OFQ up to 24 h (Rizzi et al. 2014). PWT2-SP was in vivo 3- to 10-fold more potent than SP and displayed approximately fourfold longer duration of action (from ∼5 min for SP to ∼20 min for PWT2-SP) (Ruzza et al. 2014). The in vivo behavior of PWT1-NPS is close to that of PWT2-SP. The reason for these sequence-dependent effects of PWT derivatives is at present unknown. It will be useful to apply this technology to several different peptide sequences to understand if these differences are related, for instance, to different susceptibility to proteases and/or different mechanisms by which the peptides bind and activate their receptors.

While the PWT increased the potency and the duration of action of NPS in vivo, it did not affect the peptide selectivity for NPSR. In fact, PWT1-NPS failed to produce wake-promoting effects in mice lacking the NPSR protein. This result demonstrated that the mechanism by which PWT1-NPS exerted its wake-promoting effect is the selective activation of NPSR.

In conclusion, the present study extended the use of the PWT to the NPS sequence. PWT1-NPS mimicked NPS actions in vitro and behaved in vivo as a selective, potent, and long-lasting NPSR agonist. Thus the present work, together with previous findings (Rizzi et al. 2014; Ruzza et al. 2014), demonstrated that PWT is an innovative and efficient strategy to generate interesting peptide receptors ligands. PWT1-NPS can be proposed as an innovative tool for the study of the pharmacology and the neurobiology of the NPS/NPSR system particularly for investigating those conditions in which a prolonged activation of the NPSR is desirable, that is, panic and anxiety disorders (Xu et al. 2004; Leonard et al. 2008; Rizzi et al. 2008; Pulga et al. 2012) and possibly memory impairment (Han et al. 2009, 2013; Zhao et al. 2010; Okamura et al. 2011).

## Abbreviations

ANOVA: analysis of variance
DMF: *N*,*N*-dimethylformamide
GPCR: G proteins coupled receptor
HBSS: Hank’s Balanced Salt Solution
HEK293_mNPSR_: human embryonic kidney 293 cells expressing the murine NPSR
LA: locomotor activity
N/OFQ: nociceotin/orphanin FQ
NKA: neurokinin A
NKB: neurokinin B
NPS: neuropeptide S
NPSR(−/−): NPSR knockout
NPSR(-/-): NPSR wild type
NPSR: NPS receptor
PWT: peptide welding technology
RR: righting reflex
SP: substance P
TFA: trifluoroacetic acid
